# Clinical Feasibility Studies and Potential Applications of Cone-Beam Computed Tomography Integrated in Multimodality X-Ray System for Small Animals

**DOI:** 10.3390/ani16050763

**Published:** 2026-03-01

**Authors:** Elena Mínguez-Pereira, Daniel Sanderson, Mónica Abella, Xiaolin Ye, Nerea León, Alejandro Sisniega, Juan Manuel Arco, María Isabel García-Real

**Affiliations:** 1 Department of Small Animal Medicine and Surgery, Veterinary Faculty, Complutense University of Madrid, 28040 Madrid, Spain; 2 Veterinary Teaching Hospital, Complutense University, 28040 Madrid, Spain; 3 Department of Bioengineering, Carlos III University of Madrid, 28911 Madrid, Spain; 4 Sedecal Molecular Imaging, 28119 Madrid, Spain

**Keywords:** computed tomography, cone beam, flat panel detector, multidetector, small-animal diagnostic imaging, dog, cat

## Abstract

Diagnostic imaging is essential in veterinary practice, and cone-beam computed tomography (CBCT) has emerged as a promising tool to complement radiography. This study aimed to optimize the image quality of a novel multimodality veterinary X-ray prototype integrating direct digital radiography, fluoroscopy and CBCT, and to assess its potential clinical applications, focusing on the CBCT component. The study was conducted in three phases: optimization of CBCT image quality using postmortem samples, comparison of CBCT and 16-slice multidetector CT (MDCT) images of four cadavers (two dogs and two cats), and potential clinical applications in 24 live patients. Comparative evaluation in postmortem scans revealed that CBCT achieved equal quality in 65% of bone compared to MDCT and a slightly inferior quality in 90% of soft-tissue structures using the bone reconstruction protocol, with beam hardening as the main limiting factor. Clinical validation showed that CBCT was particularly useful for identifying small fractures and mineralized structures, providing diagnostic information not clearly visible on radiographs. Integration of radiography, fluoroscopy, and CBCT in a single device facilitated workflow and allowed a more precise diagnosis in most of the patients examined with the prototype, which demonstrated promising diagnostic performance in small-animal and exotic veterinary practice.

## Simple Summary

Veterinary medicine uses advanced imaging to visualize internal structures non-invasively, allowing precise diagnosis and treatment planning without exploratory surgery. Digital radiography is accessible and provides rapid information, but its two-dimensional nature with overlapping tissues can hinder the visualization of pathologies. Computed tomography, a three-dimensional imaging technique that uses a rotating X-ray source to build volumetric images, solves the tissue overlapping. This study evaluated a prototype of a new multimodality veterinary imaging system that combines digital X-rays, real-time moving X-ray imaging (fluoroscopy), and cone-beam computed tomography in a single device. Image quality was first optimized using animal specimens and then compared with images obtained with a conventional device: a multidetector computed tomography scanner. The prototype provided excellent bone detail, with slight dark-streak artifacts caused by the limited angular range required by the open-gantry design that crucially allows animal access. Clinical use in live patients showed that this device facilitated the detection of small fractures and mineralized structures that were indistinct on conventional radiographies. By integrating multiple imaging modalities into one unit, the system improved workflow and reduced patients’ sedation time. These results indicate that this device has strong potential for enhancing diagnostic capabilities in small-animal and exotic species practice.

## 1. Introduction

Diagnostic imaging has become essential in small-animal clinical day-to-day practice, providing non-invasive tools to achieve diagnosis and to help decision-making in a wide variety of cases. Radiography is one of the most used techniques due to availability, cost and ease of use [1]. Digital radiography (DR) has largely replaced conventional radiography [2], since it improves workflow by reducing total examination time significantly [3]. DR provides almost immediate image display, better image quality and reduced repeat exposure, functions electronically and allows digital study sharing [4]. There are two main types of digital radiology acquisition: computed radiography (CR) and direct digital radiography (DDR), which is further classified into flat panel detectors (FPDs) and charge-coupled devices (CCDs) [2,5].

Despite these advantages, the diagnostic value of radiography is limited by the superimposition of complex anatomical structures. To overcome this limitation, cross-sectional imaging modalities such as computed tomography (CT) emerged. CT devices can be classified according to their acquisition geometry, with the multidetector CT (MDCT) and cone-beam CT (CBCT) being the most widely used geometries in clinics. MDCT equipment has multiple rows of detectors arranged in a semicircular array opposite to the X-ray tube and a fan-shaped beam, which is moved along the gantry while the tube-detector system rotates continuously around the patient, resulting in a helical path [6,7,8,9]. This enables the scanning of large anatomic areas with shorter study times and thinner sections [6,7], allowing quasi-isotropic spatial resolution [7,10], when using small acquisition pitch values. In small-animal veterinary medicine, MDCT has a wide variety of applications, including the evaluation of head, spine, thoracic and abdominal diseases, cancer staging, surgery planning and angiography [6,9,11,12,13]; similar applications have been described for exotic animals [14].

On the other hand, CBCT is a modality in which an X-ray tube and a flat panel detector (FPD) rotate around the patient to acquire volumetric data in a single rotation [15,16]. It has two main mechanical designs: the C-arm configuration, also called flat panel detector computed tomography (FDCT), which may operate with limited arc rotations, typically 180–240° [17], or fixed, with full 360° rotation and a closed gantry similar to the one used in MDCT, which is classically referred to as CBCT [18]. Although technically different, FDCT and CBCT are often used interchangeably in the literature [15,16].

CBCT offers excellent spatial resolution in high-contrast structures [16,17], but contrast resolution for low-contrast objects is limited by factors such as increased scatter, lower radiation dose and further limitations of the imaging chain [15,19]. It also has decreasing resolution away from the isocenter and artifacts appearing due to non-compliance with the data sufficiency condition, especially compared to MDCT [20]. In human medicine, CBCT is increasingly used as an alternative to MDCT in specific cases such as musculoskeletal trauma [21], pediatric extremity trauma [22], detection of nasal and paranasal pathologies before rhinoplasty [23], oral medicine [24], and assessment of the spine in surgery [25].

CBCT’s applications in veterinary medicine are still being explored, but similar to human medicine [15], it is mainly used in small-animal dentistry to evaluate ear and dental structures in cats [26,27] and to assess dental abnormalities in dogs and cats, demonstrating improved visualization compared with intraoral radiography [26,27]. In rabbits, CBCT has been applied both to normal dentition and dental disease, providing high-resolution images for clinical assessment [28,29]. In turtles, CBCT was used to generate a high-resolution dataset for 3D modeling and surgical/shell-repair planning [30]. Craniofacial applications in cats have included morphometric assessment of the temporomandibular joint, comparing CBCT with MDCT [31], and in standing equids for head evaluation [32]. CBCT systems have also been reported for quantitative assessment of bone mineral density in small animals [33] and to visualize carpal anatomical structures in comparison with MDCT in equids [34]. Comparison of CBCT and 64-slice MDCT for imaging canine dentoalveolar structures [35] showed the clinical superiority of the former. Additionally, it has been used in treatment planning for a variety of neoplasia types, including anal sac carcinoma and bladder cancer in dogs [36,37], and accurate targeting for radiation therapy [38]. Although preliminary, these reports for clinical CBCT use in veterinary medicine suggest a wide application range.

This study aims to provide a first preliminary clinical feasibility evaluation of a novel C-arm FPD system that integrates DDR, fluoroscopy and CBCT. This work focuses on the CBCT component of the prototype and its potential applications, aiming to optimize image quality and assess its utility in small-animal veterinary practice. The experimental design comprised three consecutive phases: phase 1 involved optimization of image quality using various postmortem samples; phase 2 compared CBCT images with those obtained from four cadavers (two cats and two dogs) using a 16-slice multidetector CT (MDCT); and phase 3 evaluated the potential clinical applications of CBCT in live patients.

## 2. Materials and Methods

This study was divided into three phases. Postmortem material was initially used to optimize image quality and protocol optimization by a biomedical engineering team (phase 1). After achieving reproducible and satisfactory results, comparative testing against a reference 16-slice MDCT scanner was conducted using cadavers by diagnostic imaging veterinarians (phase 2). Once the postmortem images’ quality was considered adequate for diagnostic purposes, CBCT studies were performed in patients of a veterinary hospital who required a CT as a part of their diagnostic protocol to evaluate image quality, feasibility, and investigate possible applications of the prototype (phase 3).

### 2.1. Study Population

**Phase 1:** Postmortem material included 101 samples of domestic, exotic and wild animals; 41 fossil material and 9 ham specimens were also scanned. The samples were provided by the University Department of Anatomy and Embriology for research purposes, with unknown health status.

This wide variety of samples aims to span a comprehensive sample of the possible species and anatomical sites that could be encountered during routine use of the system at later stages. Details of the samples used in this phase are given in Table 1.

**Phase 2:** Cadavers of 1 spayed female Andalusian Rat Terrier (Dog 1), 1 spayed female Shih-Tzu (Dog 2) and 2 intact short-haired male cats (Cat 1 and Cat 2) were used. All samples were from patients euthanized (Dog 1 cardiac tumor, Dog 2 heart failure, Cat 1 carpal tumor, Cat 2 intestinal neoplasia) and donated for research purposes. These cadavers were used solely for image quality comparison rather than anatomical description; therefore, inclusion was based on availability rather than health status.

Positioning was standardized as much as possible with the help of positioning foam and tape: Dog 1 and Cat 2 were positioned in sternal recumbency for both modalities, although hindlimb extension varied slightly between CBCT (separated) and MDCT (extended backwards) acquisitions in Dog 1. Dog 2 and Cat 1 were positioned in dorsal recumbency for hip and hindlimb acquisitions, and in sternal recumbency for the remaining examination in both CBCT and MDCT.

Two samples (Dog 2 and Cat 1) were scanned within approximately three hours after death in both CTs. The other two samples (Dog 1 and Cat 2) were frozen between MDCT and CBCT acquisition.

**Phase 3:** This phase was designed as a prospective evaluation of the prototype’s potential clinical applications in a hospital equipped with MDCT. The number of patients included was limited (24 patients), as CBCT was performed only in cases where CT was recommended but MDCT was not carried out, primarily due to financial limitations of the owners. These studies are summarized in Table 2. This phase represents a preliminary study aimed at exploring the practical clinical applications of the CBCT prototype.

The sedation protocol in dogs consisted of dexmedetomidine (Dexdomitor®, 0.5 mg/mL, Orion Pharma Animal Health, Espoo, Finland; 3 µg/kg intramuscular (IM)) combined with butorphanol (Torbugesic Vet®, 10 mg/mL, Zoetis Belgium SA, Louvain-la-Neuve, Belgium; 0.2 mg/kg IM) or, for procedures that were expected to be more painful, methadone (Semfortan®, 10 mg/mL, Dechra Veterinary Products, Bladel, The Netherlands; 0.3 mg/kg IM). Propofol (Propofol Lipuro®, 10 mg/mL, B. Braun Melsungen AG, Melsungen, Germany) was administered intravenously at 1.5 mg/kg until the desired level of sedation was achieved. This protocol served as a general guideline, although the anesthetists adjusted the doses and medication based on the patient’s clinical status and medical history. Patients underwent a fasting period of 12 h for solids and 6 h for liquids.

For rabbits, no fasting period was required since they are physiologically unable to vomit [39]. The sedation protocol consisted of midazolam (Dormazolam®, 5 mg/mL, Laboratorios Normon S.A., Madrid, Spain; 1 mg/kg IM) and sedation maintained with isofluorane (IsoVet®, 100%, Laboratorios Karizoo S.L., Madrid, Spain), whereas turtles were sedated with alfaxalone (Alfaxan®, 10 mg/mL, Jurox Pty Ltd., Rutherford, Australia; 5 mg/kg IV) and had a fasting period of 48 h.

Patient positioning varied among scans:

- Dogs (under sedation): The majority were positioned in sternal recumbency, except for the neck study, one carpus and one elbow, which were performed in lateral recumbency.
- Cat (under sedation): Sternal recumbency.
- Other domestic species: Sternal recumbency.
- Birds: The Eurasian scops owl study was performed without sedation, positioned in dorsal recumbency with minimal immobilization, using medical paper tape to secure the wings and a towel to cover the head. Additionally, the peacock was examined without sedation, positioned standing with minimal restraint due to its critical health status.

All living patients underwent CT for clinical purposes; therefore, no additional ethical approval was required. All data was handled anonymously.

### 2.2. Technical Equipment

The prototype diagnostic X-ray multimodality system used for this study integrates DR, digital fluoroscopy, and CBCT into a single device using an FPD, built on a robotic C-arm gantry (Multivet, Sedecal Molecular Imaging, Madrid, Spain). The system has three degrees of freedom (DoF), two translational DoFs to allow for positioning of the source–detector assembly at different locations along the scan table and at variable height, and a rotational DoF allowing for angular positioning of the C-arm gantry, as well as for controlled rotational scanning for CBCT image acquisition. The C-arm spans a total of 195 degrees, preventing protrusion of the arc from beneath the table, facilitating positioning of the patient. The system includes a rotational rhenium–tungsten anode X-ray tube, with a variable focal spot (06–1.2 FS), directly opposed to a 43 × 43 cm^2^ FPD with a columnar CsI scintillator and an a-Si pixel matrix, with a nominal pixel size of 0.1 mm. The C-arm gantry yielded a source-to-detector distance of 100 cm. To reduce the impact of scattered radiation, a focused anti-scatter grid with a grid ratio of 12:1 and a strip frequency of 1.85 mm^−1^ was placed immediately above the detector.

For CBCT acquisition, the center of rotation is placed closer to the detector to maximize the system field of view (FOV), yielding an isotropic FOV of size 31 × 31 × 31 cm^3^. CT images were obtained for a set of acquisition protocols with a variable number of views (196 in phases 1–2 of the study and 392 in phase 3), and with a variable X-ray technique (60 to 120 kV and 0.75 to 12 mAs, depending on patient size and anatomical region under study). Volumetric reconstruction was achieved with a variation of the Feldkamp–Davis–Kress (FDK) algorithm, resulting in an isotropic voxel size of 0.576 × 0.576 × 0.576 mm^3^ for soft-tissue protocols, and 0.278 × 0.278 × 0.278 mm^3^ for high-resolution protocols focused on assessment of high-contrast bone anatomy.

Each study involved a total of 196 projections used in phases 1 and 2, and a total of 392 projections for acquisition protocols used in phase 3 of the study. All acquisition protocols involved the acquisition of equally spaced projections over the full available angular span of the system (195 degrees) and took under 60 s for acquisition. The increase in the number of projections in phase 3 was accompanied by an equivalent reduction in per-projection mAs, yielding equal doses to the patient as those used in phases 1 and 2. Acquired projections underwent conventional offset and gain compensation, followed by compensation of gridline artifacts caused by the anti-scatter grid, and finally, by a water linearization step, used to compensate for single-material beam-hardening effects. Volumetric reconstructions were obtained with an FDK-based algorithm [40], including an apodized ramp filter and angular sampling redundancy weighting. The backprojection stage integrated a view-dependent geometrical pose description to compensate for variations in the source–detector pose as a function of gantry position. The geometrical calibration parameters were obtained in a prior calibration stage following an approach similar to the one used by Abella et al., 2018 [41].

The image quality of the CBCT prototype was compared during phase 2 with that of a conventional 16-slice MDCT scanner (Aquilion Start, Canon Medical System, Otawara, Japan). The rotation time was 0.75 s and slice thickness was 1 mm. A body image protocol for patients weighing less than 15 kg was used to scan the cadavers. The acquisition parameters ranged from 100 to 120 kV and 67.5–150 mAs, depending on the anatomical region scanned.

In all 4 postmortem specimens of phase 2, MDCT examinations were completed in a single acquisition due to the greater scanning length coverage of the system (150 cm maximum), whereas CBCT whole-body examination required three acquisitions, each covering an individual FOV equivalent to the nominal FOV size for a single rotation of the scanner (31 cm).

### 2.3. Data Collection

All images were stored in DICOM format and reviewed using RadiAnt DICOM Viewer (version 2025.2, Medixant, Poznań, Poland). For each case, the following information was collected in a spreadsheet:

- Case identification number and date of examination.
- Species and breed. Sex and age if applicable.
- Patient status (postmortem or in vivo).
- Anatomical region examined.
- Acquisition parameters (kV, mAs).

### 2.4. Image Analysis

The analysis of the images was performed by a veterinary diagnostic imaging professor with 30 years of professional experience and a PhD student with 3 years of experience in the field. Technical support was provided by a biomedical engineering team specialized in this field. The algorithms were optimized for the visualization of bone structures, but soft-tissue algorithms were in the development stage.

**Phase 1:** Image quality was initially evaluated using general qualitative criteria focusing on anatomical detail, tissue contrast and the presence of artifacts, with special attention to whether these artifacts could affect diagnostic quality.

**Phase 2:** A standardized visualization protocol was established to ensure maximum consistency in the comparison between postmortem CBCT studies and studies performed in MDCT. Five anatomical regions were evaluated, each including assessment of osseous structures (cortical and medullar evaluation) and surrounding soft tissues (subcutaneous fat when present, muscles and other relevant structures):

- Head (tympanic bulla CT transverse image):
   o Bone structures: tympanic bulla (*bulla tympanica*) wall, *septum bullae* (cats), osseus labyrinth (*labyrinthus osseus*), hyoid bone (*apparatus hyoideus*), parietal bone (*os parietale*).
   o Soft-tissue structures: mandibular lymph nodes (*lymphonodi mandibulares*).
- Thorax (accessory lung lobe CT transverse image):
   o Bone structures: ribs (*os costale*).
   o Soft-tissue structures: lung lobe differentiation, epaxial muscles (when fat tissue is present).
- Abdomen (renal pelvis of the left kidney CT transverse image):
   o Bone structures: vertebrae.
   o Soft-tissue structures: renal pelvis (*pelvis renalis*) and intra-abdominal adjacent organs (intestines (*intestina*), kidney (*renes*), spleen (*lien*) when visible), intra-abdominal fat (when present).
- Hip (coxofemoral joint CT transverse image):
   o Bone structures: acetabulum, femoral head (*caput ossis femoris*).
   o Soft-tissue structures: intrapelvic urethra, rectum.
- Distal tarsal bone row (centrodistal tarsal joint CT transverse image):
   o Bone structures: tarsal bones (*os tarsale I*, *II*, *III*, *IV*).
   o Soft-tissue structures around the bones.

Once the region of interest had been identified in both modalities, CBCT images were initially displayed using the Full Dynamic preset of the RadiAnt DICOM Viewer. This preset applies an automatic linear grayscale transformation based on the minimum and maximum pixel values present in the image, assigning pure black and pure white accordingly and redistributing intermediate attenuation values across the grayscale to optimize image contrast. For bone reconstruction MDCT images, window level (WL) and window width (WW) were manually adjusted to match the ones set for CBCT, and these images were then considered the MDCT reference standard for image quality comparison. A visual characteristic analysis was performed for bone and soft-tissue evaluation following a scoring system described by Ludewig et al. in 2010 [42], using a relative visual grading chart (VGA) shown in Table 3 to grade the studies from 1 (test image clearly superior to the reference image) to 5 (test image clearly inferior to the reference image).

**Phase 3**: A similar protocol was used in the in vivo patients, also based on the scoring system described by Ludewig et al. in 2010 [42]. A 5-point absolute grading chart (Table 4) was applied to clarify if the study had limitations for clinical use considering the primary clinical motive for performing the CT study. A score value of 1 indicates excellent image quality with no limitations for clinical use, and a score value of 5 indicates poor image quality and an unusable image.

## 3. Results

### 3.1. Phase 1

A total of 101 samples were scanned in this phase. Projections underwent Parker weighting to compensate for uneven data sampling caused by the limited span of the system and were reconstructed with an FDK-based algorithm. To compensate for the mechanical misalignments of the gantry during rotation, calibration parameters determined in a prior geometrical calibration step as in Abella et al., 2018 [41] were applied. To correct the cupping artifacts and recover contrast in soft-tissue structures, the water linearization algorithm was used.

Due to rigor mortis, proper positioning in some specimens was not possible. The main artifacts observed were decreased image uniformity and shading in regions joining high-attenuation features, attributable to residual beam hardening and scattered radiation. Increased noise across high-attenuation regions was seen in the first scanned hams. Residual gridline artifacts were also present in some studies.

**Figure 1:**
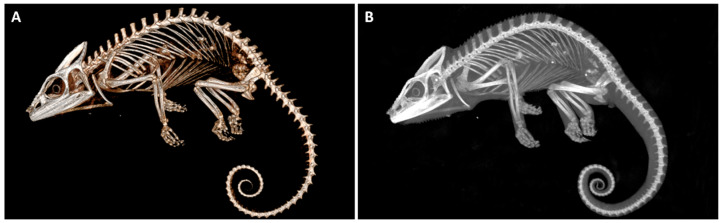
Postmortem chameleon CBCT scan showing (**A**) a lateral view of a three-dimensional (3D) volume rendering reconstruction and (**B**) sagittal Maximum-Intensity Projection (MIP).

**Figure 2:**
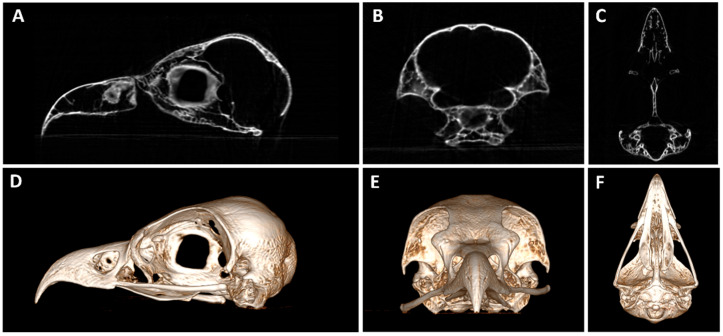
(**A**) Sagittal, (**B**) transverse and (**C**) dorsal planes of a postmortem bird skull CBCT and (**D**–**F**) their corresponding three-dimensional (3D) volume rendering reconstruction.

**Figure 3:**
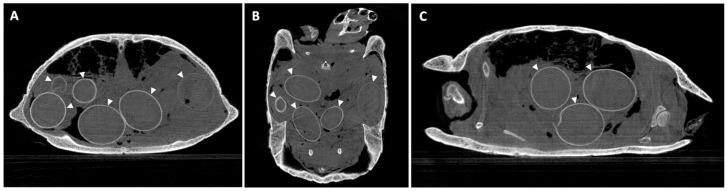
Postmortem turtle CBCT scan in (**A**) transverse, (**B**) dorsal and (**C**) sagittal planes; arrowheads show calcified egg shells.

**Figure 4:**
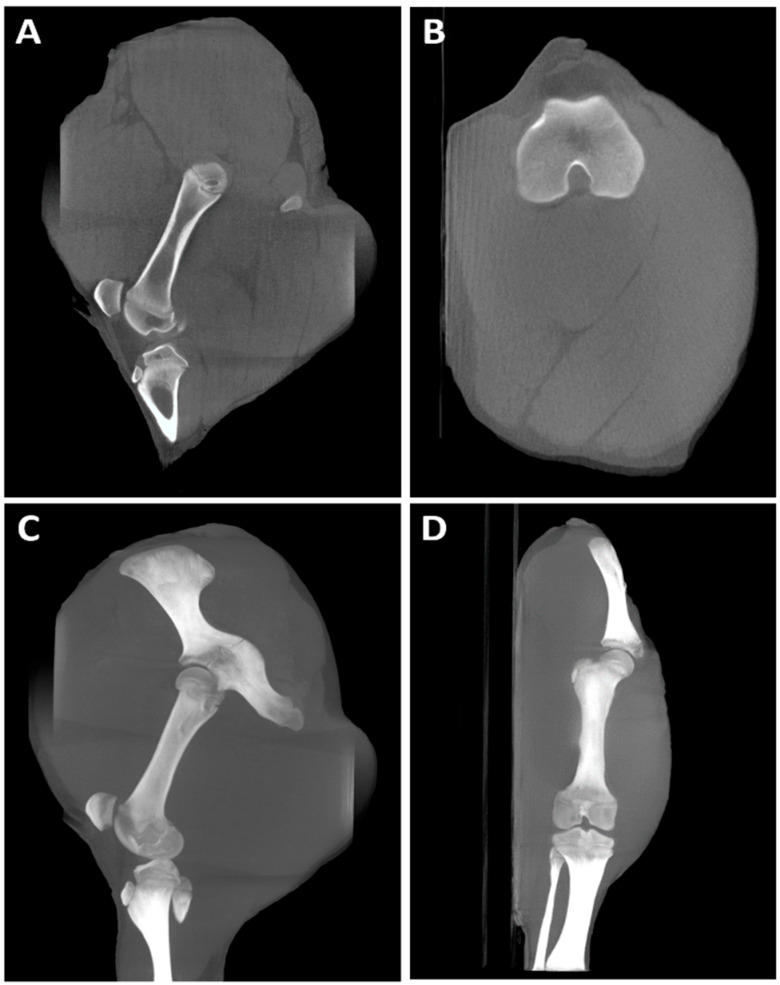
Ham CBCT scan in (**A**) sagittal and (**B**) transverse planes; (**C**) sagittal and (**D**) dorsal Maximum-Intensity Projection (MIP).

**Figure 5:**
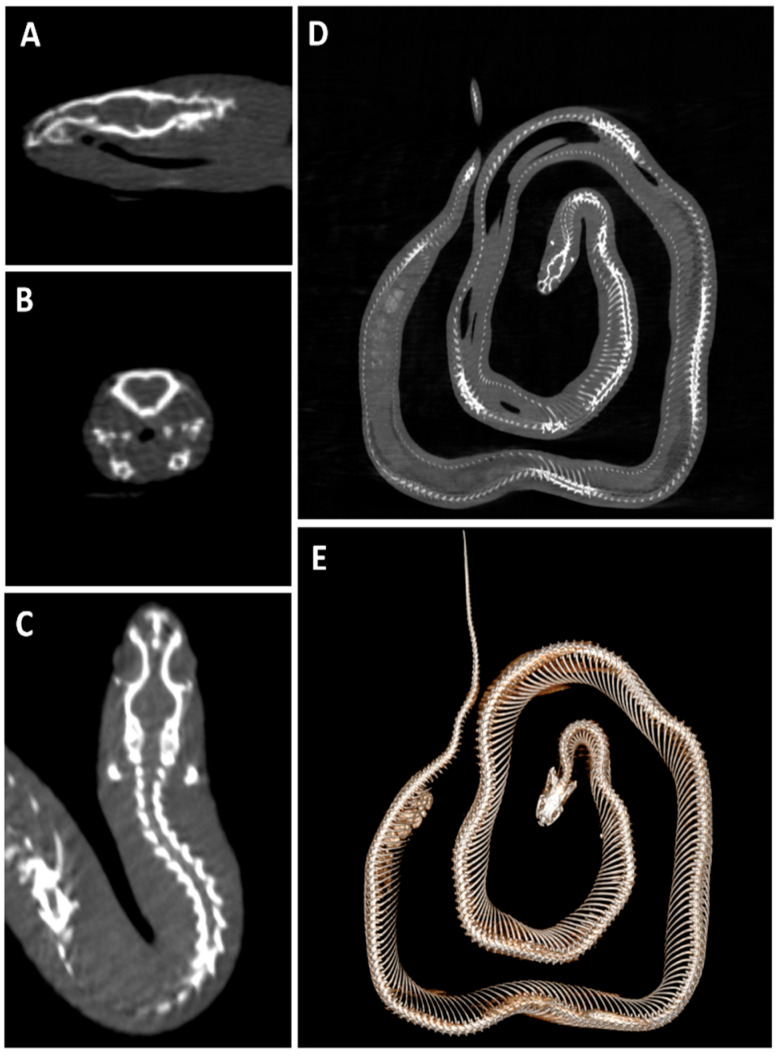
Postmortem snake CBCT scan. (**A**) Sagittal, (**B**) transverse and (**C**) dorsal planes of the head; (**D**) whole-body dorsal plane and (**E**) the corresponding three-dimensional (3D) volume rendering reconstruction.

In larger samples like hams or the horse head, four acquisitions were needed to perform the whole sample scan. Some examples of postmortem scans are shown in Figure 1, Figure 2, Figure 3, Figure 4 and Figure 5, including a chameleon (Figure 1), a bird skull (Figure 2), a turtle (Figure 3), a ham specimen (Figure 4) and a snake (Figure 5).

In this phase, the high definition of the bone structures stood out, especially in the smaller specimens, such as the head of the snake, the bird skull, and the chameleon. In contrast, the differentiation between soft tissues was limited.

### 3.2. Phase 2

Four postmortem specimens (two dogs and two cats) were examined. For each specimen, five predefined anatomical regions (head, thorax, abdomen, hip, and centrodistal tarsal row) were evaluated, including both osseous and soft-tissue components as specified in the Materials and Methods section. In total, 20 anatomical structures were analyzed.

The MDCT protocol was performed by gantry rotation around the moving bed, using the positioning tools available in the imaging system, with no need to reposition the patient in between scans. In the version of the CBCT imaging system used for this phase, multi-position scans were not fully automated, resulting in a longer scan time compared to MDCT. The increased time was mostly attributable to repositioning of the gantry in between scans and to the need to launch several scans.

**Figure 6:**
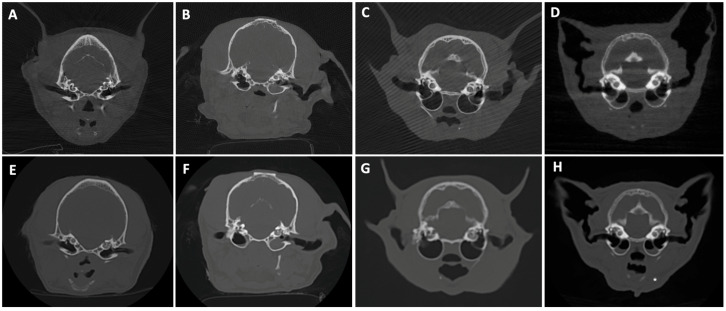
CBCT (**top**) and MDCT (**bottom**) images at the tympanic bulla level of the postmortem samples used in phase 2 of the study. (**A**,**E**) Dog 1; (**B**,**F**) Dog 2; (**C**,**G**) Cat 1; (**D**,**H**) Cat 2, with a white asterisk showing the mandibular lymph node in MDCT.

**Figure 7:**
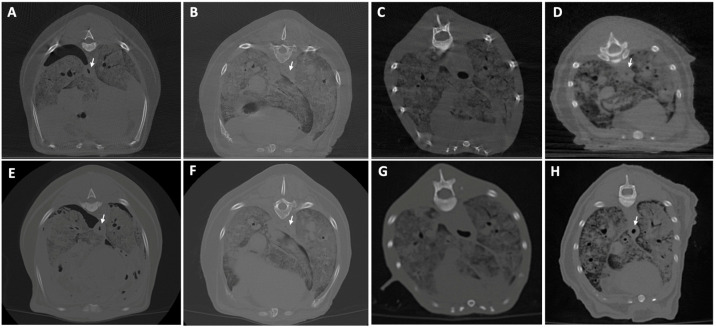
CBCT (**top**) and MDCT (**bottom**) images at the accessory lung lobe level of the postmortem samples used in phase 2 of the study. (**A**,**E**) Dog 1; (**B**,**F**) Dog 2; (**C**,**G**) Cat 1; (**D**,**H**) Cat 2. The white arrow illustrates the esophagus.

**Figure 8:**
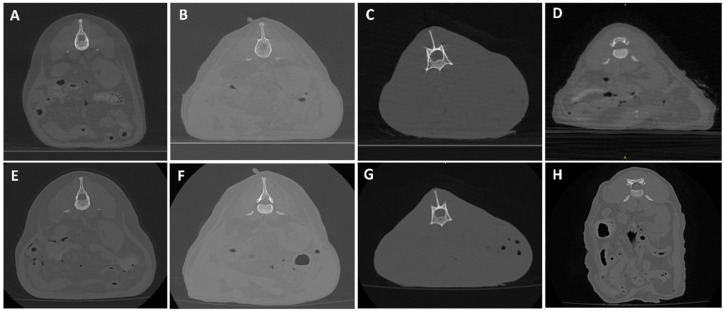
CBCT (**top**) and MDCT (**bottom**) images at the renal pelvis of the left kidney level of the postmortem samples used in phase 2 of the study. (**A**,**E**) Dog 1; (**B**,**F**) Dog 2; (**C**,**G**) Cat 1; (**D**,**H**) Cat 2.

**Figure 9:**
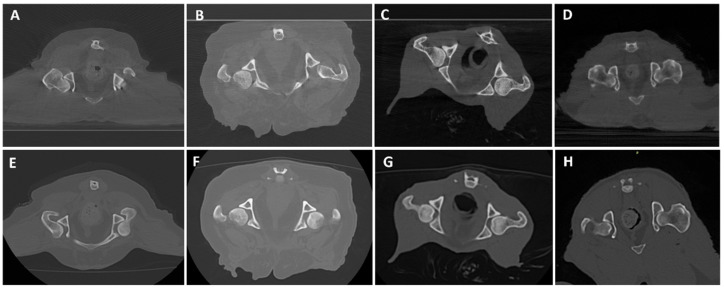
CBCT (**top**) and MDCT (**bottom**) images at the coxofemoral joint level of the postmortem samples used in phase 2 of the study. (**A**,**E**) Dog 1; (**B**,**F**) Dog 2; (**C**,**G**) Cat 1; (**D**,**H**) Cat 2.

**Figure 10:**
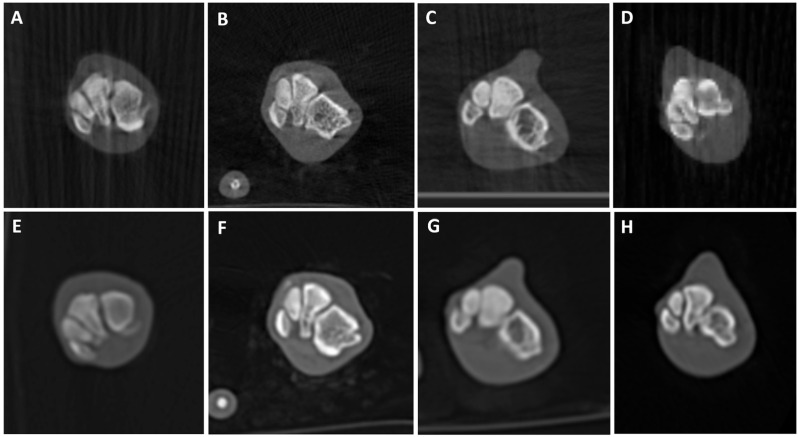
CBCT (**top**) and MDCT (**bottom**) images at the distal tarsal bones row of the postmortem samples used in phase 2 of the study. (**A**,**E**) Dog 1; (**B**,**F**) Dog 2; (**C**,**G**) Cat 1; (**D**,**H**) Cat 2.

**Figure 11:**
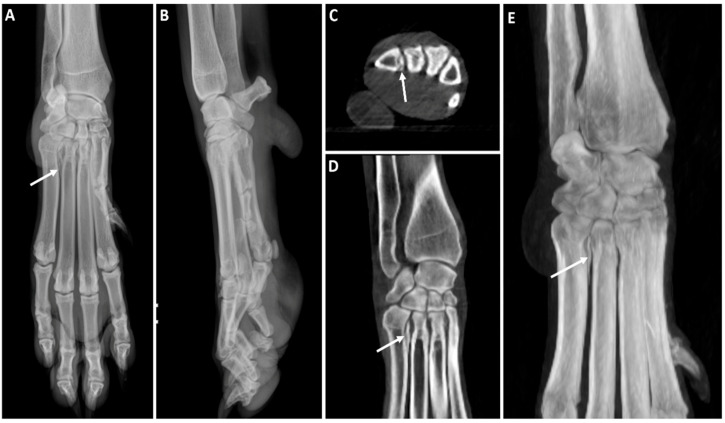
Left forelimb of a 2-year-old White Swiss Shepherd dog with acute lameness onset after falling while crossing a river. (**A**) Dorsopalmar radiograph that shows a poorly defined radiolucent line adjacent to the medial aspect of the proximal fifth metacarpal bone (white arrow). (**B**) Mediolateral radiograph where superimposition of structures does not allow assessment of the area of interest. (**C**) CBCT transverse image at the proximal metacarpal level where a clear hypoattenuating line crosses the medial aspect of the fifth metacarpal bone (white arrow). (**D**) CBCT dorsal image which shows a well-defined line fracture (white arrow). (**E**) Dorsal MIP that better illustrates the extent of the fracture (white arrow).

Figure 6, Figure 7, Figure 8, Figure 9, Figure 10 and Figure 11 illustrate the comparison between CBCT and MDCT at each landmark CT transverse image.

#### 3.2.1. Bone Evaluation

Overall examined structures in CBCT were scored as 3, which means that image quality is equal to the reference image provided by the MDCT (65%, 13/20 anatomical structures of the samples examined, panels A and E from Figure 6, Figure 7, Figure 8, Figure 9 and Figure 10, panels B and F from Figure 7, Figure 8, Figure 9 and Figure 10 and panels C and G from Figure 6, Figure 7, Figure 8 and Figure 9). Thirty percent (6/20, panels D and H from Figure 6, Figure 7, Figure 8, Figure 9 and Figure 10 and panels C and G from Figure 10) were scored as 4, where the CBCT image is somewhat inferior to MDCT. The tympanic bulla region (head) of Dog 2 proved to be slightly superior (score 2, Figure 6B) to the MDCT (5%, 1/20) (Figure 6F), as beam-hardening artifacts were less pronounced than in the other samples. These results are summarized in Table 5.

#### 3.2.2. Soft Tissue Evaluation

All structures examined in CBCT were scored as 4, slightly inferior to the reference image provided by the MDCT (95%, 19/20 anatomical structures of the samples examined; panels A–H from Figure 7, Figure 8, Figure 9 and Figure 10 and panels A–C and E–G from Figure 6), excepting the tympanic bulla region (head) of Cat 2, since it appeared to be clearly inferior to the MDCT (5%, 1/20, panels D and H from Figure 6), where the mandibular lymph nodes were visible in the latter but not in CBCT due to beam-hardening artifacts (Table 5).

The thoracic landmark was selected to evaluate the CBCT capability to delimitate tissues of different densities within a complex area that contains multiple anatomical structures. However, pulmonary assessment per se in postmortem specimens cannot emulate in vivo conditions, since the lungs collapse after death, thus eliminating normal contrast between aerated parenchyma and surrounding soft tissues. In those conditions, most of the structures were visible and well delimited except for the esophagus in Cat 1 (Figure 7C), most likely due to its poor body condition with reduced periesophageal fat, which limited its visualization in both CTs.

In the abdominal region, Dog 1 showed comparable image quality between CBCT (Figure 8A) and MDCT (Figure 8E), with minimal artifact presence and quite similar soft-tissue differentiation, probably due to the abundant intra-abdominal fat.

### 3.3. Phase 3

#### 3.3.1. Dogs (*n* = 14)

The carpus was the region most scanned in dogs (28.57%, 4/14), with the majority of studies having good image quality and minimal limitations for clinical use (score 2). In one of the cases (Figure 11), a dorsopalmar radiograph showed a poorly defined radiolucent line adjacent to the medial aspect of the proximal fifth metacarpal bone that could not be assessed in the orthogonal view. Subsequent CBCT imaging confirmed this finding.

The elbow was scanned in three cases (21.43%, 3/14), representing the second most scanned region in this preliminary dataset, and CBCT proved to be useful to assess elbow dysplasia and an atypical elbow luxation.

**Figure 12:**
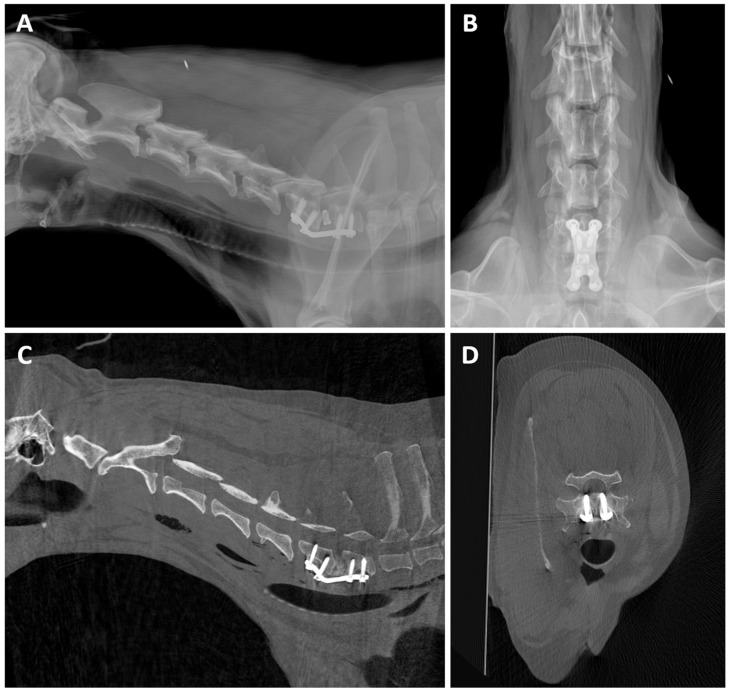
Post-surgery CBCT of an 11-year-old Weimaraner with Wobbler Syndrome. (**A**) Lateral radiograph of the cervical spine, showing a metallic cervical fusion cage between C6 and C7, along with a cervical ventral locking plate system fixed with screws. There was a suspicion that at least one of the two most cranial screws could get into the vertebral canal. (**B**) Ventrodorsal radiograph of the cervical spine, where the plate is superimposed to the vertebral bodies and does not allow assessment of the screw position. (**C**) Sagittal CBCT and (**D**) transverse CBCT images at the level of C6 show that both cranial screws fall slightly into the vertebral canal.

Spinal imaging in dogs (14.29%, 2/14) provided good image quality and allowed evaluation of vertebral bone integrity. In one of the cases (Figure 12), a lateral radiograph of the cervical spine raised suspicion that at least one of the two most cranial screws could get into the vertebral canal. Since the ventrodorsal radiograph had the plate superimposed over the vertebral bodies, it did not allow assessment of the screws. Consequently, CBCT was recommended to further assess the implant and the vertebral canal.

**Figure 13:**
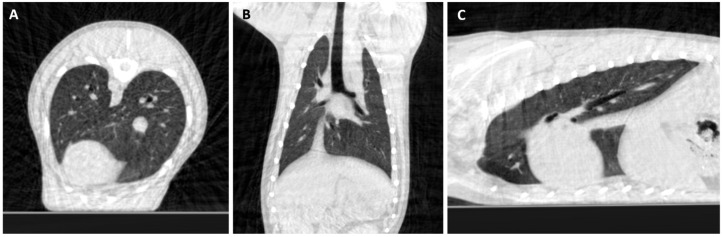
Thoracic CBCT scan of a 7-year-old Bichon Frise with a history of unproductive cough for 3 months, before tracheal lavage. (**A**) Transverse, (**B**) dorsal, (**C**) and sagittal CBCT images displayed with bone algorithm, although with window width (WW) and window level (WL) optimized for lung evaluation.

Thoracic images (14.29%, 2/14) (Figure 13) also showed sufficient performance with some limitations; image quality was slightly reduced in a tachypneic patient due to motion artifacts, but CBCT still complemented radiographic findings and provided valuable diagnostic information for lung assessment in two dogs, one with chronic cough and another for metastasis search.

**Figure 14:**
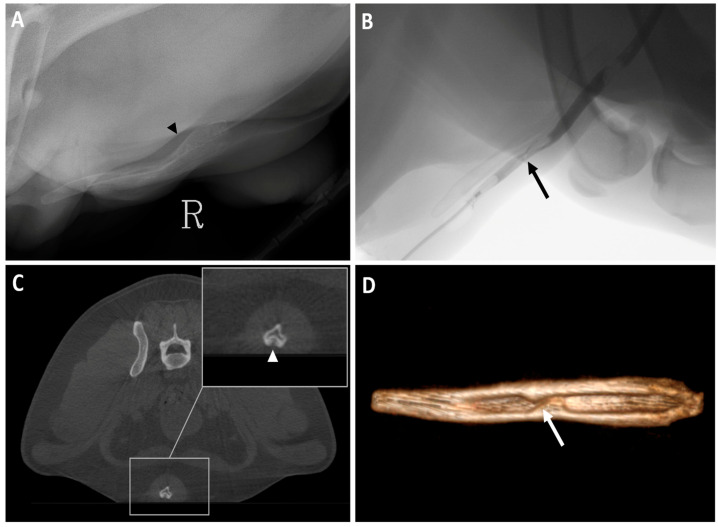
Abdominal images of a 7-year-old American Staffordshire crossbreed who presented with stranguria for one month. (**A**) Detail of a right (R) lateral radiograph of the abdomen (hindlimbs pulled cranially) zoomed to the os penis (black arrowhead), with no relevant findings. (**B**) Fluoroscopic image of retrograde urethrogram where a narrowing of the urethral lumen is identified (black arrow). (**C**) Transverse CBCT of the abdomen at S1 level, where bilateral new bone formation in the ventral aspect of the os penis is seen (white arrowhead). (**D**) CBCT three-dimensional (3D) volume rendering image of the os penis (ventral view) where the new bone formation is clearly visible (white arrow).

The abdominal CBCT study (7.14%, 1/14) (Figure 14) had good image quality and was performed following inconclusive ultrasonographic, radiographic and fluoroscopic findings to further evaluate the urethra. Lateral abdominal radiography did not reveal relevant abnormalities, and fluoroscopic retrograde urethrography identified a focal narrowing of the urethral lumen without clearly defining the underlying cause. CBCT subsequently allowed visualization of new bone formation responsible for narrowing of the penile urethral lumen.

**Figure 15:**
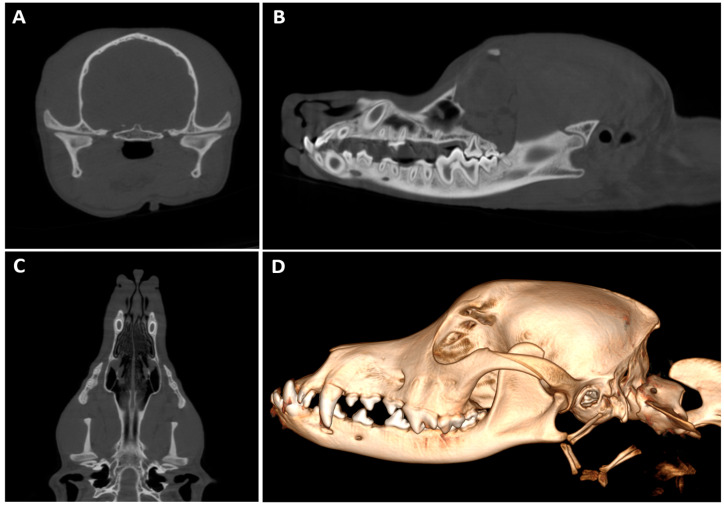
Head CBCT of a 1-year-old Andalusian Rat Terrier with impaired mastication after being hit by a car two weeks prior. (**A**) Transverse, (**B**) sagittal and (**C**) dorsal CBCT images of the temporomandibular joints, showing a good bilateral joint congruency. (**D**) CBCT three-dimensional (3D) volume rendering image of the head. Incidental findings include rostral extension of the mandible as compared to the maxilla (prognathism). The oral cavity shows complete dentition with no evident abnormalities.

Head (7.14%, 1/14) (Figure 15) and hip (7.14%, 1/14) studies were also performed. Table 6 illustrates the motive of the studies performed.

Image quality ranged from restricted to excellent using the absolute visual characteristic analysis chart. Most studies showed good image quality (50% scored 2, 7/14), followed by sufficient (28.57% scored 3, 4/14), excellent (14.29% scored 1, 2/14), and restricted image quality (7.14% scored 4, 1/14). Table 6 also specifies the VGA score for each patient.

#### 3.3.2. Cat (*n* = 1)

The single spine study showed sufficient image quality, with moderate limitations for clinical use but no substantial loss of information (score 3). The evaluation of this study was partially limited by beam-hardening artifacts from a metallic shotgun pellet in the lumbar vertebrae L6.

#### 3.3.3. Exotic Animal (*n* = 9)

**Figure 16:**
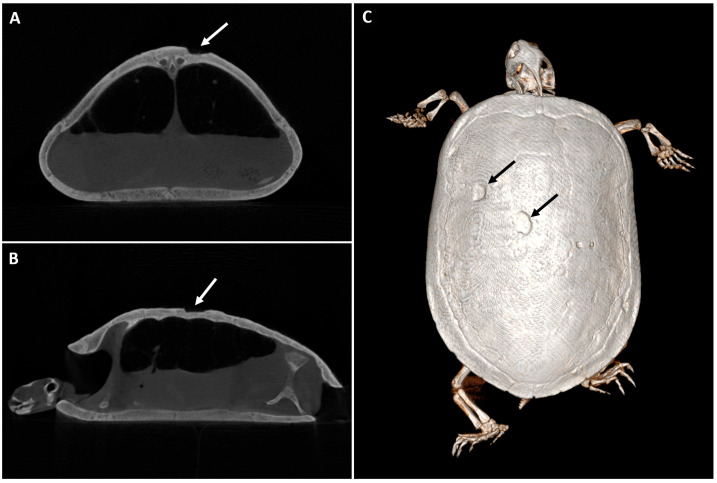
CBCT of a 10-year-old red-eared slider turtle with progressive ulceration in the shell. (**A**) Transverse and (**B**) sagittal CBCT images where a big ulcer is visible (white arrow), not compromising the lungs. (**C**) CBCT three-dimensional (3D) volume rendering image (dorsal view) that shows two shell ulcers (black arrows).

Image quality varied among patients, with 55.56% (5/9, score 3) scored as sufficient, 22.22% (2/9, score 4) as restricted and 22.22% (2/9, score 2) as good image quality. Specific species included:

- Rabbits (*n* = 3): All head CTs, scored as 3 (100%, 3/3).
- Turtles (*n* = 3): Whole-body CTs, with 66.67% (2/3) scored as 3 and 33.33% (1/3) scored as 2. Figure 16 illustrates one of the clinical cases of a turtle with ulcers in its shell.
- Rat (*n* = 1): Head CT, scored as 4 (100%, 1/1).
- Avian patients (*n* = 2): One whole-body CT graded as 2 (50%, 1/2), and one thorax scan scored as 4 (50%, 1/2).

## 4. Discussion

In the present study, optimization and validation of the image quality of a veterinary multimodality diagnostic imaging prototype that integrates direct digital radiography, digital fluoroscopy and CBCT have been performed. The present work has focused on the CBCT component of the prototype and its potential clinical applications. The experimental design comprised three consecutive phases: phase 1 involved optimization of image quality using various types of postmortem samples; phase 2 focused on the comparison between images of four cadavers (two cats and two dogs) acquired with a 16-slice MDCT and the prototype CBCT; and phase 3 explored potential clinical applications of CBCT.

Phase 1 was performed in collaboration with biomedical engineers to get the first extensive database of images from animals with different morphological characteristics. This data served to make the necessary technical adjustments before starting the next phases of the study. Fossil and food material were included in this phase to allow the engineers to adjust the prototype settings for diverse shapes and densities. While these samples were not the focus of the study, non-clinical applications of MDCT for similar materials have been reported in the literature [43,44], demonstrating the broader applicability of the imaging techniques that allow non-invasive evaluation of structures.

In phase 2, the comparison between CBCT and MDCT using canine and feline cadavers provided a more standardized preclinical context for assessing image quality. This approach is similar to the one described by Grunz et al. in 2020 [45], who compared a 3D CBCT system with high-resolution MDCT in human cadaveric elbows and found their CBCT prototype delivered superior image quality compared to the latter. Although the prototype’s hardware is different from the one used in this study, it similarly integrates direct digital radiography, fluoroscopy and CBCT in a single device. Additionally, there is a study that compares CBCT versus MDCT images of the carpal region of horses in ex vivo limbs [34], where CBCT was found to be a reliable diagnostic imaging modality for this region in equines.

A standardized protocol for image evaluation was set, selecting several anatomic landmarks: tympanic bullae for the head, accessory lung lobe for the thorax, renal pelvis of the left kidney for the abdomen, coxofemoral joints for the hip and distal tarsal bone row. Given the study design requiring assessment of representative regions that are distributed along the entire body, whole-body scans of the postmortem samples were required. Due to the limited FOV of the system, three separate CBCT acquisitions were performed, each covering a different anatomical segment. This approach increased the overall examination time compared to MDCT examinations performed on the same cadavers, where the larger longitudinal coverage of the 16-slice MDCT allowed complete evaluation in a single continuous scan.

The trabecular pattern of the osseous structures of Dog 1, Cat 1 and the remaining anatomical landmarks of Dog 2 are more clearly visible on CBCT than on MDCT. Although this would correspond to a score of 2 following the visual characteristic analysis chart, as it also shows higher spatial resolution as described in existing literature [16,17], beam-hardening artifacts still affected overall margin delineation of the bone in some regions and a score of 3 was assigned. On the other hand, 95% of soft-tissue structures showed slightly inferior quality (score 4) when imaged with CBCT in comparison to the MDCT, mainly due to beam-hardening artifacts, which produced edge distortion around dense structures, consistent with what is already described in the literature for CBCT [46].

Since the image quality of phase 2 was sufficient to reliably visualize osseous structures and most soft tissues with the specific soft-tissue algorithms in the development stage, it was considered appropriate to proceed with imaging of live patients (phase 3). The primary anticipated differences between postmortem and in vivo CBCT imaging related to potential motion artifacts in live patients, whereas beam-hardening artifacts were expected to occur similarly. Nevertheless, slight variations in image quality between live cases likely reflected the ongoing refinement of post-processing techniques during this phase. These adjustments led to a noticeable improvement in bone image quality, achieving higher spatial resolution compared to the initial examinations.

Phase 3 of the study included patients where radiography did not provide sufficient diagnostic information and CBCT was considered to provide relevant additional information. The appendicular skeleton and the spine were the most scanned regions, which is consistent with findings described in human medicine, where CBCT is commonly used for musculoskeletal system pathologies [21,22,25,47,48,49].

The carpus was the region most scanned in dogs, showing the majority of studies had good image quality and minimal limitations for clinical use (score 2). Our results confirmed that CBCT was superior to radiography in fracture detection, since it allowed the identification of a proximal metacarpal bone fracture in one case and a small acetabular avulsion fragment in the hip of another patient, with neither of them clearly seen in the previous radiographs. This correlates well with previously reported results in human medicine [21,48]. Furthermore, CBCT provided valuable information in cases involving small-size foreign bodies located in the interdigital space adjacent to the central metacarpal pad and joint subluxation.

In exotic patients, CBCT imaging performance varied across species. Most reports in the literature describe the use of ultra-high-definition CBCT systems for assessing rabbits and rat heads [28,50,51], since the small size of this structure requires higher spatial resolution. While ultra-high-definition CBCT scanners generally have restricted FOV, like the one used by Riggs et al. [28] that was 18 × 20 cm, the prototype used in this study offers a larger FOV (31 cm), enabling imaging of larger regions than the former and prioritizing multimodal workflow efficiency. Micro-CT has also been described to assess different structures in ex vivo rabbit heads [52] and in experimental settings for describing the normal abdominal and thoracic anatomy of Siberian hamsters [53,54]. However, the longer acquisition times reported (up to 45 min in the -designed micro-CT in the study by De Rycke et al. [52]) make CBCT more suitable for routine clinical use in these species. The prototype provided sufficient image quality for clinical assessment of teeth and bullae in rabbits, although with moderate limitations. In rats, image quality was restricted with some loss of information, likely due to reduced head size and lower detector resolution compared with ultra-high-definition CBCT and micro-CT described in the literature.

In avian patients, image quality ranged from sufficient to good, since the peacock exhibited motion artifacts because of tachypnea, but the owl had minimal artifacts, probably due to its lower respiratory rate. There is a lack of peer-reviewed studies for CBCT use in avian patients in contrast with the literature related to MDCT (or microCT), which has been described for assessing the skeletal system [55], in surgical planning and reproductive diseases [14].

In chelonians, two turtles showed sufficient and one good image quality; however, beam-hardening artifacts related to dense shells likely contributed to a decrease in overall image quality. To the authors’ knowledge, there is only one published reference about the use of CBCT for examination of domestic turtles [30], since most of these patients are scanned using MDCT to assess common pathologies [56] or even in specific procedures like percarapacial ovocentesis [57].

Many application-specific advantages in CBCT devices have been reported in human medicine, such as high spatial resolution [16,17], which allowed the detection of occult fractures in X-rays [21,48] and lower radiation doses compared to MDCT [22,45,58]. While no thorough dose evaluation studies were included in this work, the lower total mAs used in CBCT scans when compared to MDCT scans points to a reduction in radiation dose in CBCT imaging. The reduction in radiation dose is partly responsible for the increased noise observed in CBCT datasets for evaluation of soft-tissue structures. Although an economic analysis of purchasing a CBCT was not an objective of this study, it is a factor that has been mentioned by other authors, such as Posadzy et al. [47] who found in 2018 that CBCT was 21–30% cheaper compared to a range of different MDCT manufacturers. Additionally, in our study, we found that integration of radiography, fluoroscopy and CBCT imaging within the same device facilitated workflow due to the ease of transition between techniques in the same table, and therefore, sedation time was minimized. Furthermore, this prototype can be installed in more confined spaces than MDCT, an advantage that was also highlighted by Posadzy et al. in 2018 [47].

A clear disadvantage of the CBCT evaluated in this study is the restricted FOV which may prevent evaluation of larger anatomical regions, particularly in large-breed dogs, where sequential acquisition may be required to cover the entire region of interest, although at the expense of increased total acquisition time.

The main limitation of the present study is the low number of in vivo patients included, although the dataset acquired can give an idea of the potential clinical applications of the CBCT integrated in a multimodality X-ray device. On the other hand, software development is still in progress, so some of the results obtained in this work could be improved in the near future. In addition, studies with intravenous contrast administration should be carried out.

## 5. Conclusions

CBCT integrated in a multimodality X-ray prototype can provide adequate image quality for bone structures and sufficient quality for soft tissues in the anatomical regions examined when postmortem samples are used. For in vivo studies, CBCT showed moderate limitations in clinical image quality but preserved sufficient anatomical detail to allow consistent image interpretation in all 24 examined patients. It proved to be useful for assessing small fractures, small foreign bodies and subtle skeletal lesions in dogs, cats and exotic species, while integration with radiography and fluoroscopy facilitated workflow and minimized sedation time in patients.

## Figures and Tables

**Table 1:** Postmortem samples used in phase 1 of the study for image quality evaluation and protocol optimization of the prototype’s cone-beam computed tomography (CBCT) component.

Group Classification	Samples	Number of Samples
**Domestic animals ^1^**	Dogs	16
	Cats	3
**Exotic ^1^**	Turtles	4
	Ferret	1
	Chameleon	1
	Guinea Pig	1
	Rabbit	1
	Hedgehog	1
	Snake	1
	Chinese water dragon	1
	Bearded dragon	1
	Frog	1
	Rat	1
	Mouse	1
	Sugar Glider	1
**Wild animals ^1^**	Birds	9
	Raccoon	1
**Farm animals ^1^**	Foal	2 heads
	Horse	1 head
	Calf	1 spine, 1 rib
	Goat	1 skull
**Food samples ^2^**	Ham	9
**Fossil material (fragments) ^3^**	Wolf	22
	Hyena	16
	Cat	2
	Lion	1

^1^ Samples provided by the Department Section of Anatomy and Embriology, Veterinary Faculty, Complutense University of Madrid. ^2^ Samples provided by the Department Section of Nutrition and Food Science, Veterinary Faculty, Complutense University of Madrid. ^3^ Samples provided by the Department of Geodynamics, Stratigraphy and Paleontology, Geological Sciences Faculty, Complutense University of Madrid.

**Table 2:** Case distribution in phase 3 of the study for image quality evaluation, feasibility and possible applications of the prototype’s cone-beam computed tomography (CBCT) component.

Group Classification	Species	Anatomical Region Examined	Number of Scans
**Domestic**	Dog	Carpus	4
		Elbow	3
		Thorax	2
		Abdomen	1
		Pelvis	1
		Head	1
		Cervical spine	1
		Lumbar spine	1
	Cat	Lumbar spine	1
**Exotic**	Turtle	Whole body	3
	Rabbit	Head	3
	Rat	Head	1
**Wild**	Owl	Whole body	1
	Peacock	Thorax	1

**Table 3:** Relative visual characteristic analysis chart scoring for the cone-beam computed tomography (CBCT) and the multidetector computed tomography (MDCT) images.

Score	Interpretation
1	CBCT image clearly superior to MDCT
2	CBCT image somewhat superior to MDCT
3	CBCT image equal to MDCT
4	CBCT image somewhat inferior to MDCT
5	CBCT image clearly inferior to MDCT

**Table 4:** Absolute visual characteristic analysis chart scoring for the prototype’s cone-beam computed tomography (CBCT) component.

Score	Interpretation
1	Excellent image quality, no limitations for clinical use
2	Good image quality, minimal limitations for clinical use
3	Sufficient image quality, moderate limitations for clinical use but no substantial loss of information
4	Restricted image quality, relevant limitations for clinical use, clear loss of information
5	Poor image quality: image not usable, loss of information, image must be repeated

**Table 5:** Phase 2 visual grade analysis scoring results in the different anatomical regions examined when comparing the images of the cone-beam computed tomography (CBCT) and the multidetector computed tomography (MDCT).

Structure	Tissue	Scored as 2	Scored as 3	Scored as 4	Scored as 5
**Head**	Bone	25% (1/4)	50% (2/4)	25% (1/4)	0
	Soft Tissue	0	0	75% (3/4)	25% (1/4)
**Thorax**	Bone	0	75% (3/4)	25% (1/4)	0
	Soft Tissue	0	0	100% (4/4)	0
**Abdomen**	Bone	0	75% (3/4)	25% (1/4)	0
	Soft Tissue	0	0	100% (4/4)	0
**Hip**	Bone	0	75% (3/4)	25% (1/4)	0
	Soft Tissue	0	0	100% (4/4)	0
**Centrodistal tarsal row**	Bone	0	50% (2/4)	50% (2/4)	0
	Soft Tissue	0	0	100% (4/4)	0

**Table 6:** Summary of patients that underwent a CBCT in phase 3 of the study, where possible applications of the prototype were evaluated.

Sample	Species	Region	VGA Score	Motive of the Study	Previous Image Tests Performed
Dog 1	Canine	Abdomen	2	Urinary obstruction	X-ray, ultrasound, fluoroscopy
Dog 2	Canine	Carpus	2	Carpal hyperextension	X-rays
Dog 3	Canine	Carpus	2	Multiple mineral foreign bodies	X-rays
Dog 4	Canine	Carpus	3	Lameness ten months after being hit by a car, carpal instability	X-rays
Dog 5	Canine	Carpus	2	Possible fracture of V metacarpal bone	X-rays
Dog 6	Canine	Elbow	4	Elbow dysplasia evaluation	X-rays
Dog 7	Canine	Elbow	3	Elbow luxation	X-rays
Dog 8	Canine	Elbow	1	Elbow dysplasia evaluation	X-rays
Dog 9	Canine	Head	1	Politraumatism	X-rays
Dog 10	Canine	Hip	3	Politraumatism	X-rays
Dog 11	Canine	Spine	2	Wobbler syndrome post-surgery scan	X-rays
Dog 12	Canine	Spine	2	Osteolytic lesion in spinal process of one vertebrae	X-rays
Dog 13	Canine	Thorax	2	Coughing	X-rays
Dog 14	Canine	Thorax	3	Search for metastasis, doubtful silhouette on radiograph	X-rays
Cat	Feline	Spine	3	Metallic shotgun pellet at the level of L6	X-rays
Exotic animal 1	Rabbit	Head	3	Bilateral otitis externa	None
Exotic animal 2	Rabbit	Head	3	Dental disorders	None
Exotic animal 3	Rabbit	Head	3	Nasal discharge with obstruction of the right nasolacrimal duct	None
Exotic animal 4	Rat	Head	4	Otitis media	None
Exotic animal 5	Peacock	Thorax	4	Intrathoracic mass, chronic respiratory disease	X-rays, fluoroscopy
Exotic animal 6	Owl	Wing	2	Possible fracture	X-rays
Exotic animal 7	Turtle	Turtle	2	Shell ulceration	None
Exotic animal 8	Turtle	Turtle	3	Genitourinary disorders	None
Exotic animal 9	Turtle	Turtle	3	Limb edema	None

## Data Availability

The data presented in this study are available on request from the corresponding author.

## Abbreviations

The following abbreviations are used in this manuscript:

CBCT	Cone-beam computed tomography
CCD	Charge-Coupled Device
CR	Computed Radiography
CT	Computed Tomography
DDR	Direct Digital Radiography
DoF	Degree of Freedom
DR	Digital Radiography
FDK	Feldkamp–David–Kress
FOV	Field of View
FPD	Flat Panel Detector
FDCT	Flat Panel Detector Computed Tomography
IM	Intramuscular
MDCT	Multidetector computed tomography
MIP	Maximum-Intensity Projection
WL	Window Level
WW	Window Width
3D	Three-dimensional
